# Neighborhood disadvantage predicts health resource utilization after lumbar spine surgery: a cohort study

**DOI:** 10.1007/s00701-025-06703-4

**Published:** 2025-12-01

**Authors:** Yifei Sun, Sasha Howell, Nicholas M. B. Laskay, Lucia D. Juarez, B. Grey Vandeberg, Anil Mahavadi, Jovanna Tracz, James Mooney, Jakub Godzik

**Affiliations:** 1 Heersink School of Medicine, University of Alabama at Birmingham, Birmingham, AL USA; 2 Department of Neurosurgery, University of Alabama at Birmingham, Birmingham, AL USA; 3 Division of General Internal Medicine and Population Science, Heersink School of Medicine, University of Alabama at Birmingham, Birmingham, AL USA; 4 Department of Neurological Surgery, University of Pittsburgh, Pittsburgh, PA USA

**Keywords:** Area deprivation index, Disparities, Neighborhood disadvantage, Length of stay, Lumbar spine, Health disparities, Outcomes

## Abstract

**Background:**

Socioeconomic factors are increasingly recognized in spine surgery outcomes. Although neighborhood-level deprivation is gaining traction in health outcomes research, its impact in lumbar spine surgery remains inconclusive.

**Objectives:**

We aimed to evaluate the association between area deprivation index and 90-day unplanned readmissions, reoperations, complications, and length of stay after lumbar spine surgery.

**Methods:**

All adult patients who underwent lumbar spine procedures at a single institution between 2011 to 2023 were retrospectively identified using Current Procedural Terminology and International Classification of Diseases 9/10 codes. Geospatial analysis was used to retrieve area deprivation index, with higher indices (> 90) being a high degree of neighborhood socioeconomic disadvantage. Propensity score matching, logistic regressions, univariate comparisons, and log-rank tests were used to assess the association of neighborhood disadvantage with outcomes of interest.

**Results:**

We identified 3568 patients [median age: 64, Interquartile Range (IQR) 56 – 71]. Patients with high neighborhood disadvantage had higher odds of unplanned readmission odds [aOR (adjusted Odds Ratio) 1.77, *p* = 0.001] and emergency department admission (OR 2.2, *p* < 0.001) within 30 days. These patients also had higher odds of readmission (aOR 1.72, *p* < 0.001) and had higher odds of ED readmission odds (OR 2.01, *p* < 0.001) within 90 days as well. Patients with high neighborhood disadvantage were more likely to have prolonged hospital length of stay (aOR 1.41, *p* = 0.038) compared to controls. Patients with high neighborhood disadvantage had delayed presentation within 30 days (14.32 vs 10.11 days, *p* < 0.01) when compared to matched controls. In sub-group analysis, patients who were black, rural, under-insured, and with high comorbidity burden were more sensitive to high neighborhood disadvantage.

**Conclusions:**

Patients with high neighborhood disadvantage have independently increased readmissions and hospital lengths of stay after elective lumbar spine surgery. Neighborhood disadvantage may play a significant role in determining spine surgical outcomes.

**Supplementary Information:**

The online version contains supplementary material available at 10.1007/s00701-025-06703-4.

## Introduction

Unplanned readmissions following lumbar spine surgery cost the US healthcare system billions of dollars each year and contribute to poor patient outcomes and experiences [17, 50]. Recent literature has highlighted the important role of socioeconomic status (SES) in predicting the post-operative course of lumbar spine patients, with factors such as race, insurance status, and income predicting increased rates of unplanned healthcare utilization in literature [10, 14, 25, 26, 28, 30, 37]. However, greater resolution of SES and social determinants of health are needed to identify areas of potential intervention.

Neighborhood socioeconomic disadvantage, as captured by Area Deprivation Index (ADI) offers an effective and validated approach to socioeconomic phenotyping and risk stratification. ADI is a nationally standardized, area-level metric developed by the Health Resources Services Administration (HRSA) that captures and compares common social determinants of health at the neighborhood level [18]. One of the most widely validated and utilized metrics of socioeconomic status in health outcomes research, it has recently gained prominence after its inclusion in certain incentive models and new reimbursement adjustment models by the Centers for Medicare/Medicaid Services (CMS) [2, 5, 29, 49].

ADI captures socioeconomic deprivation in major domains of SES in domains of education, income/employment, housing and household characteristics. It provides a nationally standardized score that allows for comparison of SES that is generalizable to multiple geographic and socioeconomic regions [27]. Research has linked poor cardiovascular, diabetes, and neurosurgical outcomes with patients who live in regions of high neighborhood deprivation [2, 5, 41, 43, 49]. However, literature remains unclear as to the effect of neighborhood disadvantage on readmissions following lumbar spine surgery, with the limited studies that exist reporting conflicting results [12, 14, 43]. Additionally, the way in which neighborhood disadvantage interacts with other patient factors to predict outcomes has not been investigated.

We sought to investigate the association of neighborhood level deprivation with readmissions, complications, length of stay, and discharge patterns following elective lumbar spine surgery. We additionally sought to identify patients who were at risk of poor outcomes as mediated by neighborhood disadvantage through effect modification analysis. We hypothesized that higher neighborhood disadvantage would be associated with increased rates of readmissions, reoperations, and hospital lengths of stay after lumbar spine surgery.

## Methods

This single center retrospective cohort study was conducted with Institutional Review Board approval (IRB- 300,009,309) Patient consent was waived given the retrospective design of the study. This article was written in compliance with the STROBE (Strengthening the Reporting of Observational Studies in Epidemiology) guidelines [48]. Consent was not sought due to the retrospective nature of the study.

### Study design

We identified all patients undergoing thoraco-lumbar decompression or decompression with fusion from 2011 to 2023 using CPT (Current Procedural Terminology) and ICD (International Classification of Diseases) 9/10 codes from an institutional administrative database. Inclusion criteria were adult patients (≥ 18 years) undergoing elective procedures. Exclusion criteria included patients undergoing fusion due to traumatic etiologies to reduce confounding.

### Defining variables

Patient demographic, socioeconomic, operative details, and post-operative data for each patient were collected via retrospective review of the electronic medical record (EMR). Study variables included were age at time of surgery categorized according to standard groups (< 45, 45–54, 55–64, 65–74, and ≥ 75), race (white, African American, and other), gender (Male or Female), and insurance status which was categorized as private, public (Medicare, Medicaid, Tricare), or indigent/self-pay [35].

### Variable definition

Body mass index (BMI) at time of surgery was collected from the EMR and categorized according to current convention (< 18.5, underweight; 18.5–25, normal weight; 25–30, overweight; > 35, obesity). Elixhauser Comorbidity Indices, chosen due to demonstrated association with spine readmissions, were extracted from ICD 9/10-CM codes and calculated with Van Walraven weighting, then categorized into < 0, 0, 1–5, 6–13, 14 + [51]. Pre-operative American Society of Anesthesiologists (ASA) classification were selected as well as a validated measure of surgical risk [8, 52]. Operative characteristics such as levels fused, and surgical approach (minimally invasive vs open) were collected from the medical record. Total levels operated were divided into 1–3 levels, 3–5 levels, or 5 or more levels [24]. Distance from institution was calculated by calculating the straight-line distance in miles between the coordinates of patient address to our institution.

Post-operative variables included were unplanned readmission within 30 days [Post-operative day (POD) 1–30], unplanned readmission within 90 days (POD 1–90). Unplanned readmissions were defined as any recorded hospital admission to any department of the hospital in accordance with standard guidelines [54]. Readmissions due to trauma were excluded.

Reasons for readmissions were collected via review of the documented primary complaint and were divided into three categories; Medical, defined as any medical complication including but not limited to hypocalcemia, respiratory arrest, dyspnea, MI, post op ileus, urinary incontinence, and renal failure. Infection/Wound readmission was defined as any readmission involving infection, wound infection, wound dehiscence, or drainage from wound. The final category of readmission due to surgical complications were defined as readmissions related to complications related to the surgery, such as increased post op pain, hardware failure, or any complication that resulted in a return to OR [6, 40].

Reoperation was defined as any documented return to OR for a complication associated with the index surgery including wound revisions/debridement, hardware removals, and re-instrumentations within POD 30 and POD 90. Length of stay was measured as total length of time in hospital for the surgical encounter. Prolonged length of stay was defined as greater than 5 days [1]. Non-routine discharge was defined as discharge to any non-home facility, including other acute care hospital, rehabilitation, or death [32]. Patients who were admitted to the ED within 30 days were reviewed for further admission to another hospital service and if they were eventually reoperated on during that readmission. Further additional details regarding variable and outcomes definition can be found in the supplementary content (Supplementary Digital Content, Supplementary Methods).

Patient addresses from time of surgery were geocoded using ArcGIS software and their corresponding Federal Information Processing Standard (FIPS) code retrieved. Rural urban communicating codes (RUCA), validated measures of rurality produced by the federal government using geographic and population data, were retrieved from patient addresses, included due to its established association with socioeconomic disparity [15, 45]. ADI was retrieved from the Neighborhood Atlas dataset produced by the Center for Health Disparities Research at the University of Wisconsin School of Medicine and Public Health, with higher ADI indicating a higher level of socioeconomic disparity [18, 39]. High ADI was defined as being the 90th percentile of national ADI percentile for the purposes of analysis according to previous literature and using CMS standards.

### Statistical analysis

Categorical, binary, and ordinal variables were summarized as counts and percentages, while continuous variables were summarized as the median and interquartile range (IQR). Univariable comparison analysis was performed via utilizing the one-way analysis of variance (ANOVA), log-rank test, Pearson’s chi-squared test, Wilcoxon rank sum test, or Fisher’s exact test.

To analyze the independent effect of neighborhood deprivation on outcomes we employed nearest neighbor propensity score matching (PSM) of patients in the top 90th percentile of ADI (90–100) and patients who were not. PSM is effective in reducing confounding by balancing confounders between exposure groups to isolate the effect of the exposure of interest [12, 36, 42]. Additional details can be found in the supplement (Supplementary Digital Content, Supplementary Methods). Adjusted odds ratios (aOR) with 95% confidence intervals (CIs) are reported. Sensitivity analysis was conducted via E-value calculation. We additionally investigated effect modification of high neighborhood disadvantage for subgroups of patients with certain demographic and socioeconomic characteristics. Statistical significance was set at α = 0.05**,** and all tests for significance were two-sided. All statistical analyses were performed using R (version 4.3.1, R Foundation for Statistical Computing, Vienna, Austria)  [46].

## Results

### Patient characteristics and demographics

We identified 3568 patients [median age:64, Interquartile Range (IQR)56–71]. Of the cohort, 54% were male, 15% were black and 62% of patients had public insurance, with 2% being indigent/self-pay. The median ADI rank was 61 (IQR 37–81) and 387 (10.8%) of patients were considered having high neighborhood disadvantage. Full details regarding patient characteristics and demographics can be found in Table 1 and the supplement (Supplementary Digital Content, Table S1).

**Table 1:** Patient Characteristics and Demographics

		High ADI		
Characteristic	Overall	No	Yes	*p*-value^*2*^
	*N* = 3,568^*1*^	*N* = 3,181^*1*^	*N* = 387^*1*^	
Age	64 (56, 71)	64 (56, 71)	62 (53, 68)	< 0.001
< 45	314 (8.8%)	267 (8.4%)	47 (12%)	
45–55	509 (14%)	448 (14%)	61 (16%)	
55–65	1,035 (29%)	912 (29%)	123 (32%)	
65–75	1,287 (36%)	1,159 (36%)	128 (33%)	
>75	423 (12%)	395 (12%)	28 (7.2%)	
Gender				0.4
Female	1,641 (46%)	1,455 (46%)	186 (48%)	
Male	1,927 (54%)	1,726 (54%)	201 (52%)	
Race				< 0.001
Black	521 (15%)	356 (11%)	165 (43%)	
Other	95 (2.7%)	70 (2.2%)	25 (6.5%)	
White	2,952 (83%)	2,755 (87%)	197 (51%)	
Smoking Status				< 0.001
Current	557 (16%)	458 (14%)	99 (26%)	
Former	1,165 (33%)	1,032 (32%)	133 (34%)	
Never	1,846 (52%)	1,691 (53%)	155 (40%)	
Insurance Category				< 0.001
Indigent/Self Pay	73 (2.0%)	62 (1.9%)	11 (2.8%)	
Private	1,296 (36%)	1,192 (37%)	104 (27%)	
Public	2,199 (62%)	1,927 (61%)	272 (70%)	
ADI national rank	61 (37, 81)	57 (34, 75)	95 (93, 98)	< 0.001
Levels Operated	3.00 (2.00, 4.00)	3.00 (2.00, 4.00)	3.00 (2.00, 4.00)	0.4
1–3 levels	2,482 (70%)	2,216 (70%)	266 (69%)	
3–5 levels	862 (24%)	769 (24%)	93 (24%)	
5+ levels	224 (6.3%)	196 (6.2%)	28 (7.2%)	
Fusion	1,567 (44%)	1,385 (44%)	182 (47%)	0.2
ASA Class	3.00 (3.00, 3.00)	3.00 (3.00, 3.00)	3.00 (3.00, 3.00)	0.002
RUCA				0.3
Metropolitan	2,876 (81%)	2,570 (81%)	306 (79%)	
Micropolitan	390 (11%)	348 (11%)	42 (11%)	
Rural	96 (2.7%)	80 (2.5%)	16 (4.1%)	
Small Town	206 (5.8%)	183 (5.8%)	23 (5.9%)	
Elixhauser Comorbidity Index				< 0.001
< 0	886 (25%)	794 (25%)	92 (24%)	
0	1,151 (32%)	1,054 (33%)	97 (25%)	
1–5	690 (19%)	620 (19%)	70 (18%)	
6–13	537 (15%)	466 (15%)	71 (18%)	
14+	304 (8.5%)	247 (7.8%)	57 (15%)	
Minimally Invasive	595 (17%)	509 (16%)	86 (22%)	0.002
Distance (miles)	46 (12, 86)	45 (12, 85)	56 (6, 90)	0.007
< 50	1,919 (54%)	1,740 (55%)	179 (46%)	
50–100	1,009 (28%)	881 (28%)	128 (33%)	
>100	640 (18%)	560 (18%)	80 (21%)	
BMI category	29 (26, 34)	29 (26, 33)	31 (27, 35)	<0.001
Normal weight	633 (18%)	581 (18%)	52 (13%)	
Obese	1,650 (46%)	1,443 (45%)	207 (53%)	
Overweight	1,258 (35%)	1,131 (36%)	127 (33%)	
Underweight	27 (0.8%)	26 (0.8%)	1 (0.3%)	

^*1*^Median (Q1, Q3); *n* (%) ^*2*^ Wilcoxon rank sum test; Pearson’s Chi-squared test; Fisher’s exact test, *RUCA* Rural Urban Communication Codes; *ASA* American Society of Anesthesiologists, *BMI* Body Mass Index

### Propensity score matching

After propensity score matching, 384 patients remained in the high neighborhood disadvantage category along with 1394 matched controls. SMDs for all covariates were below 0.1 between the cohorts, indicating successful matching between the cohorts (Table 2). Full details regarding matching can be found in the supplement (Supplementary Digital Content, Figure S1, Table S2).

**Table 2:** Propensity Score Matched Cohort

	High ADI			
Characteristic	No	Yes	*p*-value^*2*^	
	*N* = 1,394^*1*^	*N* = 384^*1*^		Standardized Mean Difference
Age	63 (54, 70)	62 (53, 68)	0.087	
< 45	151 (11%)	46 (12%)		0.0231
45–55	225 (16%)	60 (16%)		–0.0447
55–65	410 (29%)	122 (32%)		–0.0085
65–75	496 (36%)	128 (33%)		0.0355
> 75	112 (8.0%)	28 (7.3%)		–0.0054
Gender			> 0.9	0.0012
Female	665 (48%)	184 (48%)		
Male	729 (52%)	200 (52%)		
Race			< 0.001	
Black	353 (25%)	162 (42%)		0.0021
Other	67 (4.8%)	25 (6.5%)		0.0077
White	974 (70%)	197 (51%)		–0.0098
Smoking Status			0.8	
Current	326 (23%)	96 (25%)		–0.0214
Former	486 (35%)	133 (35%)		0.0043
Never	582 (42%)	155 (40%)		0.0171
Insurance Category			0.4	
Indigent/Self Pay	43 (3.1%)	10 (2.6%)		–0.0079
Private	419 (30%)	104 (27%)		–0.0034
Public	932 (67%)	270 (70%)		0.0113
Levels Operated	3.00 (2.00, 4.00)	3.00 (2.00, 4.00)	0.5	
1–3 levels	965 (69%)	264 (69%)		–0.0096
3–5 levels	323 (23%)	92 (24%)		0.0072
5+ levels	106 (7.6%)	28 (7.3%)		0.0024
Fusion	633 (45%)	179 (47%)	0.7	0.017
ASA Class	3.00 (3.00, 3.00)	3.00 (3.00, 3.00)	> 0.9	–0.0258
RUCA			> 0.9	
Metropolitan	1,122 (80%)	304 (79%)		–0.0125
Micropolitan	148 (11%)	42 (11%)		0.0283
Rural	52 (3.7%)	15 (3.9%)		–0.0053
Small Town	72 (5.2%)	23 (6.0%)		–0.0105
Elixhauser Comorbidity Index			0.8	
<0	331 (24%)	91 (24%)		0.0046
0	369 (26%)	97 (25%)		0.0123
1–5	266 (19%)	69 (18%)		–0.0083
6–13	257 (18%)	71 (18%)		–0.0109
14+	171 (12%)	56 (15%)		0.0023
Minimally Invasive	272 (20%)	84 (22%)	0.3	0.0047
Distance (miles)			0.4	
<50	599 (43%)	178 (46%)		–0.0011
50–100	476 (34%)	127 (33%)		–0.0058
> 100	319 (23%)	79 (21%)		0.0069
BMI category	30 (27, 34)	31 (27, 35)	0.14	
Normal weight	204 (15%)	52 (14%)		0.0074
Obese	709 (51%)	205 (53%)		–0.0091
Overweight	475 (34%)	126 (33%)		0.0043
Underweight	6 (0.4%)	1 (0.3%)		–0.0026

^*1*^Median (Q1, Q3); *n* (%)^*, 2*^ Wilcoxon rank sum test; Pearson’s Chi-squared test; Fisher’s exact test, *RUCA* Rural Urban Communication Codes; *ASA* American Society of Anesthesiologists; *BMI* Body Mass Index

### Primary outcomes

 After matching, patients with high neighborhood disadvantage had higher odds (aOR 1.77, *p* = 0.001) of unplanned readmission and increased odds (aOR 2.2, *p* < 0.001) of ED admission in that same period (Table 3). Patients with high neighborhood disadvantage had higher odds (aOR 1.8, *p* = 0.017) for 30-day readmission due to surgical complications compared to matched patients (Table 3).

Patients with high neighborhood disadvantage had higher odds (aOR 1.72, *p* < 0.001) of unplanned readmission within 90 days of index operation and had higher odds (OR 2.01, *p* < 0.001) of ED readmission within 90 days as well. Patients with higher neighborhood disadvantage had odds (aOR 1.74, *p* = 0.014) of readmission due to medical complications and surgical complications (aOR 1.49, *p* = 0.043) compared to matched controls (Table 3).

**Table 3:** Propensity Score Matched Outcomes

	High ADI					
Characteristic	No	Yes	*p*-value^*2*^			
	*N* = 1,394^*1*^	*N* = 384^*1*^		aOR^*1*^	95% CI^*1*^	*p*-value
30-day Readmission	118 (8.5%)	54 (14%)	0.001	1.77	1.25, 2.48	0.001
30-day ED Admission	83 (6.0%)	47 (12%)	< 0.001	2.2	1.50, 3.20	< 0.001
Medical	34 (2.4%)	16 (4.2%)	0.07	1.74	0.93, 3.13	0.073
Infection/Wound	32 (2.3%)	12 (3.1%)	0.4	1.37	0.67, 2.62	0.4
Surgical	54 (3.9%)	26 (6.8%)	0.015	1.8	1.10, 2.89	0.017
Reoperation	54 (3.9%)	13 (3.4%)	0.7	0.87	0.45, 1.56	0.7
90 day Readmission	185 (13%)	80 (21%)	< 0.001	1.72	1.28, 2.29	< 0.001
90-day ED Admission	121 (8.7%)	67 (17%)	< 0.001	2.22	1.60, 3.06	< 0.001
Medical	67 (4.8%)	31 (8.1%)	0.013	1.74	1.11, 2.68	0.014
Infection/Wound	41 (2.9%)	14 (3.6%)	0.5	1.25	0.65, 2.26	0.5
Surgical	98 (7.0%)	39 (10%)	0.042	1.49	1.00, 2.19	0.043
Reoperation	78 (5.6%)	23 (6.0%)	0.8	1.07	0.65, 1.71	0.8
Non routine discharge	162 (12%)	57 (15%)	0.089	1.33	0.95, 1.83	0.09
Prolonged Hospital Length of Stay	153 (11%)	57 (15%)	0.038	1.41	1.01, 1.95	0.038

^*1*^Median (Q1, Q3); *n* (%) ^*2*^ Wilcoxon rank sum test; Pearson’s Chi-squared test; Fisher’s exact test, *ED* Emergency Department; *aOR* adjusted Odds Ratio

### Secondary outcomes

 After matching, patients with high neighborhood disadvantage were more likely to have prolonged hospital length of stay (aOR 1.41, *p* = 0.038), and had longer median times to presentation within 30 days (14.32 vs 10.11 days, *p* < 0.01) when compared to matched controls. The median time to 90-day readmission did not differ between patients with high neighborhood disadvantage and matched controls (21.9 vs 18.8 days, *p* = 0.6) (Table 4).

**Table 4:** Median times to Presentation of Matched Cohort

		Median Time to Readmission (days)	Lower 95% CI	Upper 95%CI	*p*-value
30-day Readmission	Overall	10.9	9.15	13.8	0.01
High ADI	No	10.11	8.92	13.07	
	Yes	14.32	8.57	18.93	
90 day Readmission	Overall	19.1	17.4	22.2	0.55
High ADI	No	18.76	15.61	20.64	
	Yes	21.88	18.15	27.06	

### ED admission outcomes

 Of the patients who were admitted to the ED within 30 days, patients with high neighborhood disadvantage were less likely to be eventually admitted to another hospital service (38% vs 57%, = 0.045) and were also less likely to be reoperated in that same readmission (13% vs 31%, *p *= 0.018). This was not seen at 90 days between groups (Table 5). There were no differences in acute post-operative complications between groups (Supplementary Digital Content, Table S3). Sensitivity analysis displayed robustness (Supplementary Digital Content, Fig. S2).

**Table 5:** ED Admission Outcomes

	High ADI		
Characteristic	No	Yes	*p*-value^*2*^
	*N* = 83^*1*^	*N* = 47^*1*^	
30-day ED Admission Result			0.045
Admitted to Other Service	47 (57%)	18 (38%)	
Not Admitted to Other Service	36 (43%)	29 (62%)	
Reason			
Medical	27 (33%)	16 (34%)	0.9
Infection/Wound	17 (20%)	7 (15%)	0.4
Surgical	40 (48%)	24 (51%)	0.8
Reoperation	26 (31%)	6 (13%)	0.018
Characteristic	No	Yes	
	*N* = 121^*1*^	*N* = 67^*1*^	
90-day ED Admission Result			0.084
Admitted to Other Service	61 (50%)	25 (37%)	
Not Admitted to Other Service	60 (50%)	42 (63%)	
Reason			
Medical	51 (42%)	27 (40%)	0.8
Infection/Wound	26 (21%)	10 (15%)	0.3
Surgical	61 (50%)	34 (51%)	>0.9
Reoperation	34 (28%)	14 (21%)	0.3

^*1*^ *n* (%); Median (Q1, Q3) ^*2*^ Fisher’s exact test; Pearson’s Chi-squared test; Wilcoxon rank sum test, *ED* Emergency Department

### Effect modification

After matching, subgroup analyses were performed to assess effect modification for 30-day readmission by race, insurance type, rurality, and comorbidity burden. Black patients (OR 2.77, *p* < 0.001), patients classified as Indigent/Self-Pay (OR 4.63, *p* = 0.028) or public insurance (OR 1.72, *p* = 0.007), and patients with RUCA classifications of metropolitan (OR 1.83, *p* = 0.001) or micropolitan (OR 2.54, 95% CI 1.07–5.36, *p* = 0.021) with high neighborhood disadvantage had higher odds to be readmitted compared to other patients. Those with high neighborhood disadvantage and with Elixhauser scores of 1–5 (OR 2.75, *p* = 0.001), 6–13 (OR 3.15, *p* < 0.001), and ≥ 14 (OR 2.35, *p* = 0.018) had significantly elevated risk of 30-day readmission compared to other patients (Table 6).

**Table 6:** Effect Modification of Neighborhood Disadvantage and other Socioeconomic variables

Characteristic	OR	95% CI	*p*-value
Race			
White	1.09	0.63, 1.79	0.8
Black	2.77	1.78, 4.20	< 0.001
Other	1.47	0.35, 4.34	0.5
Insurance type			
Indigent/Self Pay	4.63	0.99, 16.9	0.028
Private	1.68	0.89, 2.96	0.086
Public	1.72	1.14, 2.53	0.007
RUCA			
Metropolitan	1.83	1.25, 2.63	0.001
Micropolitan	2.54	1.07, 5.36	0.021
Rural	0.77	0.04, 3.89	0.8
Small Town	0.49	0.03, 2.37	0.5
Elixhauser Score			
< 0	1.19	0.54, 2.30	0.6
0	0.59	0.20, 1.34	0.3
1–5	2.75	1.43, 4.97	0.001
6–13	3.15	1.70, 5.54	<0.001
14+	2.35	1.10, 4.59	0.018

*RUCA* Rural-Urban Commuting Area

## Discussion

Social determinants of health have been shown to play significant roles in health outcomes. Literature has demonstrated the impact that race, income, and insurance can play on readmission and reoperations rates in spine surgery [14, 30, 37]. Recently, neighborhood level disadvantage has gained attention as a novel area level metric that effectively captures a patient’s social determinants of health and effectively models health outcomes [2, 5, 29, 49]. Additionally, neighborhood disadvantage has been announced to be included in upcoming models for reimbursement utilized by the CMS, highlighting its increased relevance [29]. However, the effect of neighborhood disadvantage in lumbar spine surgery outcomes remains unclear.

Gordon et al. [12] investigated the effect of neighborhood deprivation on 1–2 level lumbar fusions, finding that even though patients with high neighborhood deprivation had increased odds of 90-day ER utilization, they had lower odds of 90-day readmissions overall. However, analysis by Ng et al.[34] found that high neighborhood disadvantage was associated with increased rates of 30- and 90-day readmissions, reoperations, and ED utilization in a cohort of 1–2 level lumbar fusions. Similarly, analysis by Hagan et al.[14] did not find that high neighborhood disadvantage was associated with increased odds of readmissions following lumbar decompression.

We found that high neighborhood level socioeconomic deprivation was independently associated with increased rates and odds of 30-day readmissions and 90-day readmissions, as well as 30-day ED admissions and 90-day ED admissions. Of the patients who were readmitted to the ED within 30 days, patients with high neighborhood deprivation were also less likely to be readmitted to another service or require reoperations compared to matched controls.

### Readmissions

Several potential driving mechanisms may contribute to the association of neighborhood disadvantage and readmissions. High neighborhood disadvantage has been correlated with poor education levels and health literacy in spine patients. In an analysis by Lans et al.[22], spine surgery patients who had limited health literacy were found to have higher neighborhood disadvantage. Low health literacy may contribute to increased risks of wound complications and implant issues, due to poor wound care or understanding of post-discharge recovery plans, leading to increased readmissions [20, 23].

Low health literacy may also contribute to increased healthcare utilization due to lack of education of post operative complications, leading to increased potentially preventable and unnecessary readmissions [3, 4, 47]. This is supported by our evidence, in which highly disadvantaged patients who were readmitted to the ED within 30 days were less likely to be admitted to another inpatient service and less likely to return to the OR in that same encounter. This suggests that patients with high neighborhood disadvantage were generally discharged with complaints that were handled within the ED, suggesting fewer complex situations. Though it is possible that this observation may be attributable to provider bias or hospital admission preferences, strong matching of covariates between groups suggests effective control for any measured or unmeasured confounders, lending strength to this evidence.

High neighborhood disadvantage may also suggest poor access to community-level healthcare, leading to increased rates of complications as well as delayed detection of complications [16, 31, 38]. Low socioeconomic status has been shown to be associated with lower rates of established primary care and difficulties accessing care [31]. A study by Kirby et al. [19] found that high neighborhood socioeconomic disadvantage was associated with decreased chances of having consistent care and lowered likelihood of having preventative services independent of the relative supply of physicians, highlighting the significant role of neighborhood disadvantage in receiving adequate healthcare.

Patients who lack care may be at greater risk of having complications and may be delayed in their presentation for these complications as well. This is also supported by the fact that patients with high neighborhood disadvantage had longer median times to readmission within 30 days when compared to matched controls. This observation may also potentially highlight transportation issues in patient with high degrees of disadvantage [7, 44]. Though distance from institution was well balanced between the matched cohorts, difficulty with securing childcare, and securing safe transportation to the hospital may be barriers to care captured by neighborhood disadvantage, evidenced by the delayed presentation for 30 day readmission.

### Hospital length of stay

Patients with high neighborhood disadvantage were found to have increased likelihood of having prolonged hospital length of stay when compared to matched controls, suggesting that social determinants of health play a role with discharge difficulties and post-operative course while in the hospital. This finding may be attributed to comorbidity optimization at time of surgery. Patients with higher neighborhood disadvantage are known to have difficulties accessing preventative care and have generally more and poorly controlled comorbidities, contributing to poor recovery [9, 21]. Additionally, patients with high neighborhood deprivation may have lower levels of social support, transportation issues, or unstable home environments, which may contribute to increased hospital lengths of stay [27, 53].

### Effect modification

Our interaction terms also highlighted that the effects of high neighborhood disadvantage are particularly pronounced in patients of black race, low insurance coverage, high comorbidity burden, and patients living in metropolitan or micropolitan RUCA regions. These patients are particularly sensitive to the detrimental effects of neighborhood disadvantage and had disproportionately increased odds of readmission following lumbar spine surgery compared to other patients. This identifies a particularly high-risk cohort that may benefit the most from an institution-wide intervention.

### Interventions

Neighborhood disadvantage is primarily calculated with 4 primary domains of education, employment/income, housing quality, and household characteristics such as transportation, offering us a framework with which to understand these findings and design interventions [27].

Given the strong association of health literacy as a driver of poor outcomes predicted by neighborhood deprivation, targeted education efforts in patients with high neighborhood disadvantage may improve readmission rates. For example, enhanced education efforts leveraging advance practice providers implemented by Naylor et al.[33] was found to reduce readmissions at 6 weeks in both medical and surgical patients. A study by Glendenning-Napoli et al.[11] found that a community-based case management program utilizing a multidisciplinary team effectively reduced acute outpatient encounter and inpatients admissions while increasing primary care visits for patients with chronic diseases in a large academic medical center. Additionally, programs such as the PRONTO (PROgram for Non-emergency TranspOrtation) target transportation barriers in disadvantaged patients, and may improve post-op access to care [13].

Significantly, ADI provides important value by integrating multiple domains of socioeconomic disadvantage into a single, standardized, census-tract–level score. ADI reflects structural risks that may not be evident when examining race, income, or insurance alone. Because it is nationally standardized and generalizable, ADI can be consistently applied across populations and health systems, making it well suited for large-scale research, predictive modeling, and real-time identification of high-risk groups to guide clinical and policy interventions for neurosurgical spine populations.

Out data suggests that neighborhood disadvantage may be an important prognostic marker for a patient’s post-operative course and may be key in predicting patient experience, surgical metrics, cost, and potentially surgical outcomes. Further research is needed to determine the relationship and evaluate methods for optimization. Spine surgeons should consider the impact of social determinants on postoperative course and evaluate possible opportunities for pre-operative optimization.

### Limitations

Our study is limited due to its single center retrospective design. However, our institution is the primary tertiary referral center for several states in the region, thus potentially mitigating selection bias. ADI is an area-level measure of neighborhood disadvantage that is estimated from patient block group, which may not be a complete capture of patient socioeconomic status. However, our results further validate the prognostic value of ADI in lumbar spine surgery patients. Lack of patient-reported outcomes (PROs) such as ODI or pain scores limit our analysis to objective measures such as readmission rates and hospital lengths of stay, which may not fully capture patient post-operative course. Future studies should validate the effect of neighborhood deprivation on PROs and surgical outcomes in prospectively enrolled, multicenter cohorts.

## Conclusion

High neighborhood disadvantage is independently associated with increased odds of 30- and 90-day readmission, 30 and 90 day ED readmission, and prolonged length of stay. Patients with high neighborhood deprivation who were readmitted to the ED within 30 days were less likely to be admitted to another service or receive reoperations in that visit. Patients of black race, who are underinsured, reside in metropolitan/micropolitan regions, and have high comorbidity burden are particularly sensitive to these effects. These results validate the association of neighborhood deprivation and lumbar spine surgical outcomes and may be a target of intervention to improve outcomes.

## Supplementary Information

Below is the link to the electronic supplementary material.

Supplementary file1 (DOCX 222 KB)

## Data Availability

Data can be made available upon reasonable request.

## Abbreviations

ADI: Area Deprivation Index
ASA: American Society of Anesthesiologists
RUCA: Rural-Urban Commuting Area Code
HRSA: Health Resources Services Administration
CMS: Centers for Medicare/Medicaid Services
EMR: Electronic Medical Record
ED: Emergency department
FIPS: Federal Information Processing Standard
SES: Socioeconomic Status
