# Supplementary material for: Neighborhood disadvantage predicts health resource utilization after lumbar spine surgery: a cohort study
Source: Acta Neurochir (Wien). 2025 Dec 1;167(1):301. doi: 10.1007/s00701-025-06703-4 (PMC12669313; doi:10.1007/s00701-025-06703-4)
Supplement: Supplementary file 1 — Supplementary file1 (DOCX 222 KB) [file 701_2025_6703_MOESM1_ESM.docx]

**SUPPLEMENTARY DIGITAL CONTENT**

**Supplementary Methods**

Acute post-operative complications include infection, hardware failure, dural tear, post-op respiratory failure, post operative acute kidney injury (AKI), post-operative hematoma, deep vein thrombosis/pulmonary embolism (DVT/PE), new onset mental status change, any diagnosed nerve damage, post op ileus or urinary retention, respiratory complications such as pneumonia, cardiovascular complications including post-operative myocardial infarction, other general complications otherwise undefined, and death.^8,9^

Matching

Patients were matched in a 4:1 ratio with a 0.1 caliper using the nearest neighbor method. Matching was conducted on age, gender, race, smoking history/status, insurance type, levels operated, operation type (decompression only vs fusion), ASA class, RUCA code, Elixhauser Comorbidity Index, surgical approach (minimally invasive vs open), distance from institution, and BMI at time of surgery. Matching quality was assessed by ensuring the standardized mean difference was less than 0.1 and visually assessed via a love plot (**Supplement, Figure S1**). Patients who were missing baseline data were excluded from analysis.

**Figure S1.** Love Plot for Propensity Score Matching Demonstrating Successful Match

**Figure S2.** E-Value Bias Plots

**Table S1.** Patient Characteristics and Demographics Unmatched

|  |  | **High ADI** | |  |
| --- | --- | --- | --- | --- |
| **Characteristic** | **Overall** | **No** | **Yes** | **p-value***^2^* |
|  | N = 3,568*^1^* | N = 3,181*^1^* | N = 387*^1^* |  |
| Age | 64 (56, 71) | 64 (56, 71) | 62 (53, 68) | <0.001 |
| <45 | 314 (8.8%) | 267 (8.4%) | 47 (12%) |  |
| 45-55 | 509 (14%) | 448 (14%) | 61 (16%) |  |
| 55-65 | 1,035 (29%) | 912 (29%) | 123 (32%) | |
| 65-75 | 1,287 (36%) | 1,159 (36%) | 128 (33%) | |
| >75 | 423 (12%) | 395 (12%) | 28 (7.2%) | |
| Gender |  |  |  | 0.4 |
| Female | 1,641 (46%) | 1,455 (46%) | 186 (48%) | |
| Male | 1,927 (54%) | 1,726 (54%) | 201 (52%) | |
| Race |  |  |  | <0.001 |
| Black | 521 (15%) | 356 (11%) | 165 (43%) | |
| Other | 95 (2.7%) | 70 (2.2%) | 25 (6.5%) | |
| White | 2,952 (83%) | 2,755 (87%) | 197 (51%) | |
| Smoking Status | |  |  | <0.001 |
| Current | 557 (16%) | 458 (14%) | 99 (26%) |  |
| Former | 1,165 (33%) | 1,032 (32%) | 133 (34%) | |
| Never | 1,846 (52%) | 1,691 (53%) | 155 (40%) | |
| Insurance Category | |  |  | <0.001 |
| Indigent/Self Pay | 73 (2.0%) | 62 (1.9%) | 11 (2.8%) | |
| Private | 1,296 (36%) | 1,192 (37%) | 104 (27%) | |
| Public | 2,199 (62%) | 1,927 (61%) | 272 (70%) | |
| ADI national rank | 61 (37, 81) | 57 (34, 75) | 95 (93, 98) | <0.001 |
| Levels Operated | 3.00 (2.00, 4.00) | 3.00 (2.00, 4.00) | 3.00 (2.00, 4.00) | 0.4 |
| 1-3 levels | 2,482 (70%) | 2,216 (70%) | 266 (69%) | |
| 3-5 levels | 862 (24%) | 769 (24%) | 93 (24%) |  |
| 5+ levels | 224 (6.3%) | 196 (6.2%) | 28 (7.2%) | |
| Fusion | 1,567 (44%) | 1,385 (44%) | 182 (47%) | 0.2 |
| ASA Class | 3.00 (3.00, 3.00) | 3.00 (3.00, 3.00) | 3.00 (3.00, 3.00) | 0.002 |
| RUCA |  |  |  | 0.3 |
| Metropolitan | 2,876 (81%) | 2,570 (81%) | 306 (79%) | |
| Micropolitan | 390 (11%) | 348 (11%) | 42 (11%) |  |
| Rural | 96 (2.7%) | 80 (2.5%) | 16 (4.1%) | |
| Small Town | 206 (5.8%) | 183 (5.8%) | 23 (5.9%) | |
| Elixhauser Comorbidity Index | | |  | <0.001 |
| <0 | 886 (25%) | 794 (25%) | 92 (24%) |  |
| 0 | 1,151 (32%) | 1,054 (33%) | 97 (25%) |  |
| 1-5 | 690 (19%) | 620 (19%) | 70 (18%) |  |
| 6-13 | 537 (15%) | 466 (15%) | 71 (18%) |  |
| 14+ | 304 (8.5%) | 247 (7.8%) | 57 (15%) |  |
| Minimally Invasive | 595 (17%) | 509 (16%) | 86 (22%) | 0.002 |
| Distance (miles) | 46 (12, 86) | 45 (12, 85) | 56 (6, 90) | 0.007 |
| <50 | 1,919 (54%) | 1,740 (55%) | 179 (46%) | |
| 50-100 | 1,009 (28%) | 881 (28%) | 128 (33%) | |
| >100 | 640 (18%) | 560 (18%) | 80 (21%) |  |
| BMI_category | 29 (26, 34) | 29 (26, 33) | 31 (27, 35) | <0.001 |
| Normal weight | 633 (18%) | 581 (18%) | 52 (13%) |  |
| Obese | 1,650 (46%) | 1,443 (45%) | 207 (53%) | |
| Overweight | 1,258 (35%) | 1,131 (36%) | 127 (33%) | |
| Underweight | 27 (0.8%) | 26 (0.8%) | 1 (0.3%) |  |
| Complications All | 394 (11%) | 348 (11%) | 46 (12%) | 0.6 |
| Infection | 156 (4.4%) | 135 (4.2%) | 21 (5.4%) | 0.3 |
| Hardware Failure | 249 (7.0%) | 226 (7.1%) | 23 (5.9%) | 0.4 |
| Dural Tear | 2 (<0.1%) | 1 (<0.1%) | 1 (0.3%) | 0.2 |
| Respiratory Failure | 15 (0.4%) | 14 (0.4%) | 1 (0.3%) | >0.9 |
| Hematoma | 32 (0.9%) | 29 (0.9%) | 3 (0.8%) | >0.9 |
| AKI | 26 (0.7%) | 23 (0.7%) | 3 (0.8%) | 0.8 |
| DVT/PE | 21 (0.6%) | 18 (0.6%) | 3 (0.8%) | 0.5 |
| Altered Mental Status/Delirium | 23 (0.6%) | 21 (0.7%) | 2 (0.5%) | >0.9 |
| Nerve Damage | 16 (0.4%) | 12 (0.4%) | 4 (1.0%) | 0.087 |
| Death | 3 (<0.1%) | 3 (<0.1%) | 0 (0%) | >0.9 |
| 30-day Readmission | 322 (9.0%) | 268 (8.4%) | 54 (14%) | <0.001 |
| 30-day ED admission | 218 (6.1%) | 171 (5.4%) | 47 (12%) | <0.001 |
| 30-day ED Outcome | | |  | 0.03 |
| Admitted to Other Service | 114 (52%) | 96 (56%) | 18 (38%) |  |
| Not Admitted to Other Service | 104 (48%) | 75 (44%) | 29 (62%) |  |
| Medical | 79 (2.2%) | 63 (2.0%) | 16 (4.1%) | 0.007 |
| Infection/Wound | 77 (2.2%) | 65 (2.0%) | 12 (3.1%) | 0.2 |
| Surgical | 168 (4.7%) | 142 (4.5%) | 26 (6.7%) | 0.048 |
| Reoperation | 141 (4.0%) | 128 (4.0%) | 13 (3.4%) | 0.5 |
| 90-day Readmission | 504 (14%) | 424 (13%) | 80 (21%) | <0.001 |
| 90-day ED admission | 310 (8.7%) | 243 (7.6%) | 67 (17%) | <0.001 |
| 90-day ED Outcomne | | |  | 0.07 |
| Admitted to Other Service | 146 (47%) | 121 (50%) | 25 (37%) |  |
| Not Admitted to Other Service | 164 (53%) | 122 (50%) | 42 (63%) |  |
| Medical | 167 (4.7%) | 136 (4.3%) | 31 (8.0%) | 0.001 |
| Infection/Wound | 94 (2.6%) | 80 (2.5%) | 14 (3.6%) | 0.2 |
| Surgical | 280 (7.8%) | 241 (7.6%) | 39 (10%) | 0.084 |
| Reoperation | 215 (6.0%) | 192 (6.0%) | 23 (5.9%) | >0.9 |
| Length of Stay (days) | 2.00 (1.17, 3.00) | 2.00 (1.17, 3.00) | 2.00 (1.27, 4.00) | <0.001 |
| Length of Surgery (min) | 163 (104, 245) | 162 (103, 242) | 176 (113, 269) | 0.002 |
| Non Routine Discharge | | |  | 0.004 |
| Non-Routine Discharge | 375 (11%) | 318 (10.0%) | 57 (15%) |  |
| Routine Discharge | 3,193 (89%) | 2,863 (90%) | 330 (85%) | |
| *^1^* Median (Q1, Q3); n (%) *^2^* Wilcoxon rank sum test; Pearson’s Chi-squared test; Fisher’s exact test | | | | |

**Table S2.** Match Balance Metrics

| Balance Measures | | | |
| --- | --- | --- | --- |
|  |  | Type | Diff.Adj |
| distance |  | Distance | 0.0002 |
| age_cat_<45 |  | Binary | 0.0231 |
| age_cat_>75 |  | Binary | -0.0054 |
| age_cat_45-55 |  | Binary | -0.0447 |
| age_cat_55-65 |  | Binary | -0.0085 |
| age_cat_65-75 |  | Binary | 0.0355 |
| GENDER_M |  | Binary | 0.0012 |
| race_cat_Black |  | Binary | 0.0021 |
| race_cat_Other |  | Binary | 0.0077 |
| race_cat_White |  | Binary | -0.0098 |
| smoking_status_current |  | Binary | -0.0214 |
| smoking_status_former |  | Binary | 0.0043 |
| smoking_status_never |  | Binary | 0.0171 |
| insurance_cat_Indigent/Self | Pay | Binary | -0.0079 |
| insurance_cat_Private |  | Binary | -0.0034 |
| insurance_cat_Public |  | Binary | 0.0113 |
| levels_operated_category_1-3 | levels | Binary | -0.0096 |
| levels_operated_category_3-5 | levels | Binary | 0.0072 |
| levels_operated_category_5+ | levels | Binary | 0.0024 |
| instrumented |  | Binary | 0.017 |
| ASA_CLASS |  | Contin. | -0.0258 |
| RUCA_adj_Metropolitan |  | Binary | -0.0125 |
| RUCA_adj_Micropolitan |  | Binary | 0.0283 |
| RUCA_adj_Rural |  | Binary | -0.0053 |
| RUCA_adj_Small Town |  | Binary | -0.0105 |
| weighted_ecm_category_<0 |  | Binary | 0.0046 |
| weighted_ecm_category_0 |  | Binary | 0.0123 |
| weighted_ecm_category_1-5 |  | Binary | -0.0083 |
| weighted_ecm_category_6-13 |  | Binary | -0.0109 |
| weighted_ecm_category_14+ |  | Binary | 0.0023 |
| minimally_invasive |  | Binary | 0.0047 |
| dist_cat_<50 |  | Binary | -0.0011 |
| dist_cat_>100 |  | Binary | -0.0058 |
| dist_cat_50-100 |  | Binary | 0.0069 |
| BMI_category_Normal weight |  | Binary | 0.0074 |
| BMI_category_Obese |  | Binary | -0.0091 |
| BMI_category_Overweight |  | Binary | 0.0043 |
| BMI_category_Underweight |  | Binary | -0.0026 |
| Sample sizes |  |  |  |
| Control | Control | Treated |  |
| All | 3181 | 387 |  |
| Matched (ESS) | 385 | 384 |  |
| Matched (Unweighted) | 1394 | 384 |  |
| Unmatched | 1787 | 3 |  |

**Table S3.** Acute Post Operative Complications Propensity Score Matched

|  | High ADI | |  |
| --- | --- | --- | --- |
| **Characteristic** | **No** | **Yes** | **p-value***^2^* |
|  | N = 1,394*^1^* | N = 384*^1^* |  |
| Complications All | 159 (11%) | 46 (12%) | 0.8 |
| Infection | 64 (4.6%) | 21 (5.5%) | 0.5 |
| Hardware Failure | 103 (7.4%) | 23 (6.0%) | 0.3 |
| Dural Tear | 0 (0%) | 1 (0.3%) | 0.2 |
| Respiratory Failure | 7 (0.5%) | 1 (0.3%) | >0.9 |
| Hematoma | 16 (1.1%) | 3 (0.8%) | 0.8 |
| AKI | 13 (0.9%) | 3 (0.8%) | >0.9 |
| DVT/PE | 9 (0.6%) | 3 (0.8%) | 0.7 |
| Altered Mental Status/Delirium | 15 (1.1%) | 2 (0.5%) | 0.6 |
| Nerve Damage | 5 (0.4%) | 4 (1.0%) | 0.11 |
| Death | 1 (<0.1%) | 0 (0%) | >0.9 |
| *^1^* Median (Q1, Q3); n (%) *^2^* Fisher’s exact test | | | |

**Table S4.** Components of ADI

| **Education** | **Income** | **Housing** | **Household Characteristics** |
| --- | --- | --- | --- |
| % Population aged 25 years or older with less than 9 years of education  % Population aged 25 years or older with at least a high school diploma  % Employed population aged 16 years or older in white-collar occupations | Median family income in US dollars  Income disparity  % Families below federal poverty level  % Population below 150% of federal poverty level  % Civilian labor force population aged 16 years and older who are unemployed | Median home value in US dollars  Median gross rent in US dollars  Median monthly mortgage in US dollars  % Owner-occupied housing units  % Occupied housing units without complete plumbing | % Single-parent households with children younger than 18  % Households without a motor vehicle  % Households without a telephone  % Households with more than 1 person per room |

**SUPPLEMENTAL DIGITAL CONTENT REFERENCES**

1. van Walraven C, Austin PC, Jennings A, Quan H, Forster AJ. A Modification of the Elixhauser Comorbidity Measures Into a Point System for Hospital Death Using Administrative Data. *Medical Care*. 2009;47(6):626-633. doi:10.1097/MLR.0b013e31819432e5

2. Ledesma JA, Tran K, Lambrechts MJ, et al. Short-Segment versus Long-Segment Spinal Fusion Constructs for the Treatment of Adult Degenerative Scoliosis: A Comparison of Clinical Outcomes. *World Neurosurg*. Mar 2023;171:e611-e619. doi:10.1016/j.wneu.2022.12.069

3. (YNHHSC/CORE) YNHHSCCfORE. *Hospital-Wide All-Cause Unplanned Readmission Measure Final Technical Report*. 2024.

4. Bernatz JT, Anderson PA. Thirty-day readmission rates in spine surgery: systematic review and meta-analysis. *Neurosurgical Focus FOC*. 01 Oct. 2015 2015;39(4):E7. doi:<https://doi.org/10.3171/2015.7.FOCUS1534>

5. Sivaganesan A, Zuckerman S, Khan I, et al. Predictive Model for Medical and Surgical Readmissions Following Elective Lumbar Spine Surgery: A National Study of 33,674 Patients. *Spine*. 2019;44(8):588-600. doi:10.1097/brs.0000000000002883

6. Adogwa O, Elsamadicy AA, Fialkoff J, et al. Effect of employment status on length of hospital stay, 30-day readmission and patient reported outcomes after spine surgery. *J Spine Surg*. Mar 2017;3(1):44-49. doi:10.21037/jss.2017.03.08

7. Mummaneni PV, Bydon M, Knightly JJ, et al. Identifying patients at risk for nonroutine discharge after surgery for cervical myelopathy: an analysis from the Quality Outcomes Database. *Journal of Neurosurgery-Spine*. Jul 2021;35(1):25-33. doi:10.3171/2020.11.Spine201442

8. Schoenfeld AJ, Ochoa LM, Bader JO, Belmont PJ, Jr. Risk Factors for Immediate Postoperative Complications and Mortality Following Spine Surgery: A Study of 3475 Patients from the National Surgical Quality Improvement Program. *JBJS*. 2011;93(17)

9. Yadla S, Malone J, Campbell PG, et al. Early complications in spine surgery and relation to preoperative diagnosis: a single-center prospective study: Clinical article. *Journal of Neurosurgery: Spine SPI*. 01 Sep. 2010 2010;13(3):360-366. doi:<https://doi.org/10.3171/2010.3.SPINE09806>
